# The Mediating Role of Kynurenine Pathway Metabolites on the Relationship Between Inflammation and Muscle Mass in Oldest–Old Men

**DOI:** 10.1093/geroni/igaf122.2059

**Published:** 2025-12-31

**Authors:** Megan Hetherington-Rauth, Eileen Johnson, Eugenia Migliavacca, Lisa Langsetmo, Russell Hepple, Terence Ryan, Luigi Ferrucci, Denis Breuillé

**Affiliations:** Champalimaud Foundation, Lisbon, Portugal; California Pacific Medical Center Research Institute, San Francisco, California, United States; Nestlé Research, Lausanne, Vaud, Switzerland; University of Minnesota, Minneapolis, Minnesota, United States; University of Florida, Gainesville, Florida, United States; University of Florida, Gainesville, Florida, United States; National Institutes of Health, Gaithersburg, Maryland, United States; Nestlé Research, Lausanne, Switzerland

## Abstract

Tryptophan (TRP) metabolites along the kynurenine (KYN) pathway (KP) have been found to influence muscle. Proinflammatory cytokines are known to stimulate the degradation of TRP down the KP. Given that both inflammation and KP metabolites have been connected with loss of muscle, we assessed the potential mediating role of KP metabolites on inflammation and muscle mass in older men. Five hundred and five men (85.0 ± 4.2 years) from the Osteoporotic Fractures in Men cohort study with measured D3-creatine dilution (D3Cr) muscle mass, KP metabolites, and inflammation markers (C-reactive protein [CRP], alpha-1-acid glycoprotein [AGP] and a subsample [n = 305] with interleukin [IL-6, IL-1β, IL-17A] and tumor necrosis factor-α [TNF-α]) were included in the analysis. KP metabolites and inflammatory markers were measured using liquid chromatography-tandem mass spectrometry and immunoassays, respectively. 23%-92% of the inverse relationship between inflammatory markers and D3Cr muscle mass was mediated by KP metabolites (indirect effect p < .05). 3-hydroxyanthranilic acid (3-HAA), quinolinic acid (QA), TRP, xanthurenic acid (XA), KYN/TRP, 3-hydroxykynurenine (3-HK)/3-HAA, QA/3-HAA, and nicotinamide (NAM)/QA mediated the AGP relationship. 3-HAA, QA, KYN/TRP, 3-HK/XA, HKr ratio, 3-HK/3-HAA, QA/3-HAA, and NAM/QA mediated the CRP. KYN/TRP, 3-HK/XA, and NAM/QA explained the relationship for IL-6 and 3-HK/XA and QA/3-HAA for TNF-α. No mediation effect was observed for the other cytokines (indirect effect p > .05). KP metabolites, particularly higher ratios of KYN/TRP, 3-HK/XA, 3-HK/3-HAA, QA/3-HAA, and a lower ratio of NAM/QA, mediated the relationship between inflammation and low muscle mass. Our preliminary cross-sectional data suggest that interventions to alter D3Cr muscle mass may focus on KP metabolites rather than inflammation per se.

